# CMR-Derived Global Longitudinal Strain and Left Ventricular Torsion as Prognostic Markers in Dilated Cardiomyopathy

**DOI:** 10.3390/jcdd12090340

**Published:** 2025-09-04

**Authors:** Alexandru Zlibut, Michael Bietenbeck, Lucia Agoston-Coldea

**Affiliations:** 1 Department of Internal Medicine, Iuliu Hatieganu University of Medicine and Pharmacy, 400012 Cluj-Napoca, Romania; 2 Division of Cardiovascular Imaging, Department of Cardiology I, University Hospital Muenster, 48149 Münster, Germany; 3 Department of Radiology, Affidea Hiperdia Diagnostic Imaging Center, 400012 Cluj-Napoca, Romania; 4 2nd Department of Internal Medicine, Emergency County Hospital, 400012 Cluj-Napoca, Romania

**Keywords:** dilated cardiomyopathy, cardiovascular magnetic resonance, global longitudinal strain, left ventricular torsion, myocardial fibrosis, late gadolinium enhancement, risk stratification, feature tracking

## Abstract

Background: Non-ischemic dilated cardiomyopathy (DCM) is a heterogeneous myocardial disease associated with variable progression and an increased risk of major adverse cardiovascular events (MACEs). Cardiovascular magnetic resonance (CMR) allows the comprehensive evaluation of myocardial structure, function, and fibrosis. This prospective study aimed to assess the prognostic value of CMR-derived global longitudinal strain (GLS) and left ventricular (LV) torsion in patients with DCM. Methods: We prospectively enrolled 150 patients with newly diagnosed non-ischemic DCM and 100 age- and sex-matched healthy controls. All participants underwent standardized CMR protocols including cine imaging, late gadolinium enhancement (LGE), and feature-tracking analysis for myocardial deformation. LV volumes, ejection fraction (LVEF), GLS, and LV torsion were quantified. The primary endpoint was the first occurrence of MACE, defined as cardiac death, sustained ventricular arrhythmia, or heart failure hospitalization. The median follow-up was 33 months. Results: Compared to controls, DCM patients had significantly impaired LV function and myocardial mechanics: lower LVEF (35.1% vs. 65.2%, *p* < 0.001), reduced GLS (−9.2% vs. −19.7%, *p* < 0.001), and diminished LV torsion (1.04 vs. 1.95 °/cm, *p* < 0.001). GLS ≤ −8.6% was independently associated with increased MACE risk (adjusted hazard ratio [HR]: 1.09; 95% confidence interval [CI]: 1.01–1.61; *p* < 0.01). Similarly, reduced LV torsion predicted adverse events (adjusted HR: 1.37; 95% CI: 1.03–1.81; *p* < 0.01). The presence of LGE (42% of patients) further stratified risk (HR: 2.86; 95% CI: 1.48–12.52; *p* < 0.001). Conclusions: CMR-derived GLS and LV torsion are strong, independent predictors of adverse outcomes in DCM. Their integration into routine imaging protocols enhances risk stratification beyond conventional metrics such as LVEF and LGE. These findings support the use of myocardial deformation analysis in the comprehensive evaluation of patients with DCM.

## 1. Introduction

Dilated cardiomyopathy (DCM), a leading cause of heart failure and sudden cardiac death, represents a major healthcare burden. DCM is defined as a progressive left ventricular (LV) dilation and systolic dysfunction in the absence of significant coronary artery disease or abnormal loading conditions [1,2]. The clinical heterogeneity and lack of proper prognostication parameters underscore the need for advanced imaging tools to improve these gaps and to guide therapeutic decision-making. Cardiovascular magnetic resonance (CMR) has become the gold standard imaging technique in terms of volumetric assessment and myocardial tissue characterization, also offering high reproducibility and superior accuracy when compared to conventional echocardiography [3].

While studies have shown the significant role of echocardiography-based strain parameters in risk stratification and prognosis prediction in patients with DCM [4,5] emerging evidence endorse the potential ability of CMR-Feature Tracking (CMR-FT)-derived deformation parameters such as global longitudinal strain (GLS) in the prediction of outcomes across the spectrum of cardiovascular diseases, including hypertension and acute myocarditis [6,7]. GLS enables the early detection of subclinical systolic dysfunction, often before a decline in LV ejection fraction (LVEF) becomes apparent, thus improving risk stratification even in patients with preserved LVEF [8,9]. When assessed by CMR, GLS benefits from superior spatial resolution and endocardial border delineation, ensuring reliable and reproducible deformation measurements [10].

Left ventricular torsion (LV torsion), defined as the net difference between apical and basal rotation, reflects the helical architecture of myocardial fibers and has emerged as a sensitive marker of LV mechanics. Impaired LV torsion has been associated with adverse remodeling and larger infarct size following myocardial infarction [11], whereas preserved torsional dynamics correlate with improved exercise performance and overall cardiovascular function [12].

Myocardial fibrosis, a key component of pathological remodeling in DCM, directly influences both strain and torsional parameters. Fibrotic replacement of functional myocardium disrupts myocardial fiber orientation and contractile synchronicity, leading to measurable reductions in GLS and LV torsion [13,14]. The extent and spatial distribution of late gadolinium enhancement (LGE)—a surrogate for focal fibrosis—is closely associated with impaired deformation patterns, reinforcing the mechanistic link between structural damage and global myocardial performance [15,16]. Furthermore, LGE positivity identifies patients at increased risk for malignant arrhythmias and death, and this prognostic capacity is further enhanced when combined with deformation metrics such as GLS and LV torsion [17]. Recent evidence also highlights the added value of diffuse fibrosis quantification via extracellular volume (ECV) mapping in explaining subclinical functional impairment, underlining the benefit of integrative CMR phenotyping [18,19].

This article aims to evaluate the prognostic utility of CMR-derived GLS and LV torsion in patients with DCM, focusing on their role in identifying high-risk individuals and refining risk prediction beyond traditional parameters.

## 2. Methods

### 2.1. Study Population

We conducted a prospective, observational study on 150 patients with non-ischemic dilated cardiomyopathy (DCM) and 100 age- and sex-matched healthy volunteers, recruited between October 2020 and November 2024 at the Department of Internal Medicine, Iuliu Hațieganu University of Medicine and Pharmacy, Cluj-Napoca, Romania. DCM diagnosis was established according to current CMR criteria, using a standardized protocol [20]. LV dilation was defined as a LV end-diastolic volume index (LVEDVi) > 97 mL/m^2^, along with global systolic dysfunction expressed by LVEF < 45% on CMR. Only patients without significant coronary artery disease—defined as no prior myocardial infarction, revascularization, or ≥ 50% stenosis on invasive or CT coronary angiography—were eligible [21]. All participants underwent thorough clinical, biological, and imaging assessments. Exclusion criteria comprised significant valvular disease, persistent atrial fibrillation or frequent ventricular ectopy, uncontrolled hypertension (systolic BP > 160 mmHg or diastolic BP > 100 mmHg), and any known infiltrative, hypertrophic, arrhythmogenic, or peripartum cardiomyopathy. We also excluded individuals with systemic inflammatory, autoimmune, or malignant disease, as well as those with contraindications to CMR (e.g., implanted pacemakers, severe claustrophobia, or reduced renal function with glomerular filtration rate (GFR) < 30 mL/min/1.73 m^2^) [22].

The control group consisted of 100 healthy volunteers without cardiovascular disease, as confirmed by clinical examination, resting electrocardiogram (ECG), echocardiography, and CMR. Subjects with hypertension, diabetes, dyslipidemia, or any chronic systemic condition were not included in the control cohort. The study was approved by the Ethics Committee of Iuliu Hațieganu University of Medicine and Pharmacy (decision number 257/30.06.2021) and adhered to the Declaration of Helsinki. All participants provided written consent before inclusion.

### 2.2. CMR Assessment

CMR was performed in all participants using a 1.5 Tesla scanner (Magnetom Altea, Siemens Healthineers, Erlangen, Germany) with breath-hold acquisition, according to current guidelines for cardiac imaging [23]. Two independent level III CMR readers, blinded to all clinical data, analyzed the images. Cine imaging was acquired using steady-state free precession (SSFP) sequences in standard long-axis (2-, 3-, and 4-chamber) and contiguous short-axis views covering the entire LV. Typical imaging parameters included a TR of 3.6 ms, a TE of 1.8 ms, a flip angle of 60°, a slice thickness of 6 mm, a field-of-view of 360 mm, and a reconstructed temporal resolution of 25–40 ms.

**Figure 1:**
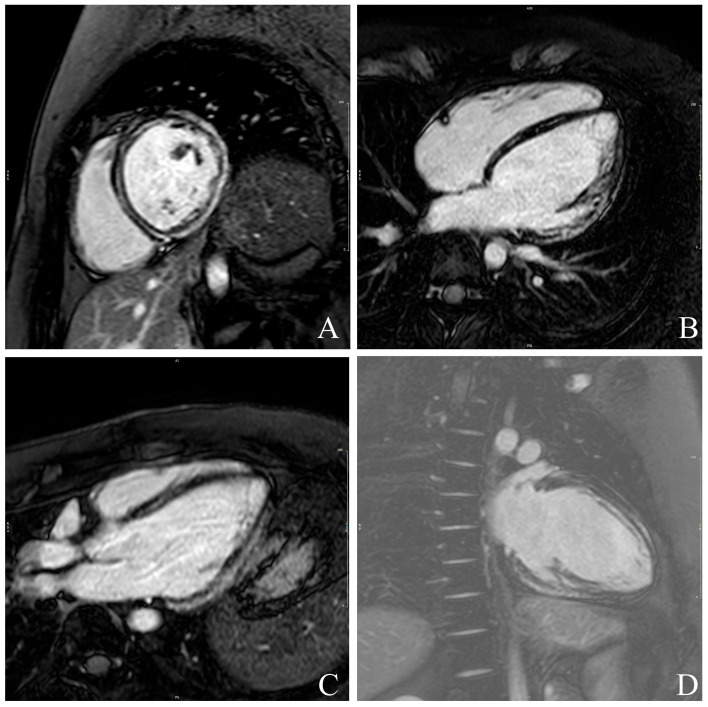
Example of late gadolinium enhancement (LGE) quantification in all four standard cardiac views: short-axis- (**A**), 4-Chamber- (**B**), 3-Chamber (**C**) and 2-Chamber (**D**)-views. Hyperenhanced myocardial regions are automatically delineated and quantified relative to total myocardial mass, allowing assessment of scar burden and distribution.

LGE was assessed 10 min after intravenous administration of 0.2 mmol/kg gadolinium-based contrast agent Gadoterate meglumine (Clariscan, GE Healthcare) using phase-sensitive inversion recovery sequences. Inversion time was adjusted per patient to optimize nulling of normal myocardium. LGE distribution was evaluated in accordance with the American Heart Association 17-segment model, excluding segment 17 [24]. The extent of LGE was expressed both as absolute mass (grams) and as a percentage of the total LV mass. LGE quantification was performed using cvi42 version 6.0.0 software (Circle Cardiovascular Imaging, Calgary, Canada), and inter- and intra-observer reproducibility were assessed in a random sample of cases (Figure 1). LGE quantification was performed using a ≥5 SD threshold above the mean signal intensity of remote, visually normal myocardium, complemented by the Full Width at Half Maximum (FWHM) technique. The total LGE volume was calculated as the sum of LGE areas across all slices multiplied by slice thickness. Global and regional LV function was derived from short-axis SSFP cine images. LV end-diastolic volume (LVEDV), end-systolic volume (LVESV), LVEF, and myocardial mass were measured and indexed to body surface area. Epicardial and endocardial borders were semi-automatically traced at both end-diastole and end-systole using a dedicated post-processing platform (Syngo Virtual Cockpit, Siemens Healthineers, Erlangen, Germany).

**Figure 2:**
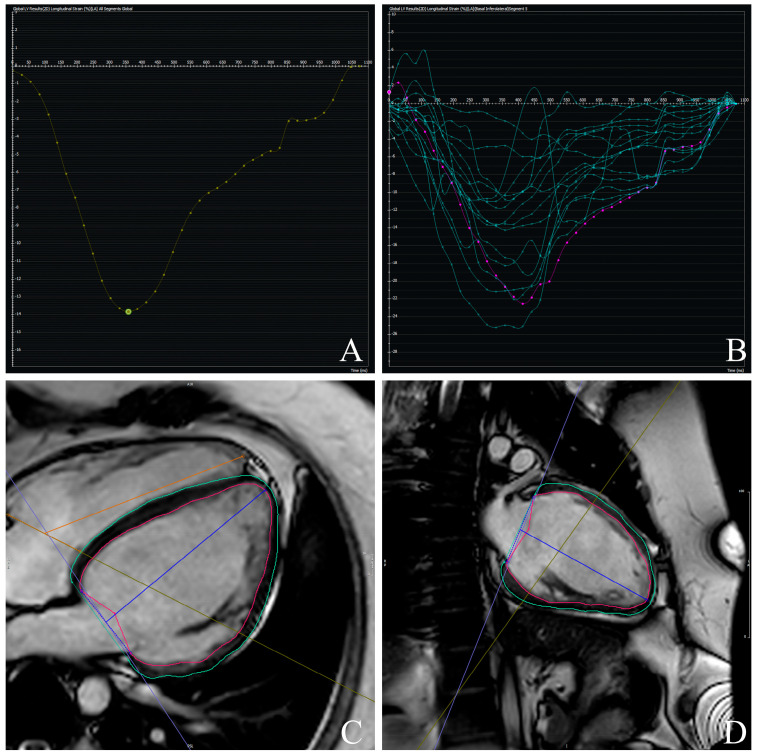
Example of left ventricular global longitudinal strain (GLS) (**A**,**B**) assessment using feature-tracking cardiac magnetic resonance (FT-CMR). Endocardial and epicardial contours are manually delineated in end-diastolic frames and automatically propagated throughout the cardiac cycle: 4-Chamber- (**C**) and 2-Chamber-Views (**D**). Myocardial deformation curves are then generated, and GLS is derived as the peak systolic longitudinal strain value.

**Figure 3:**
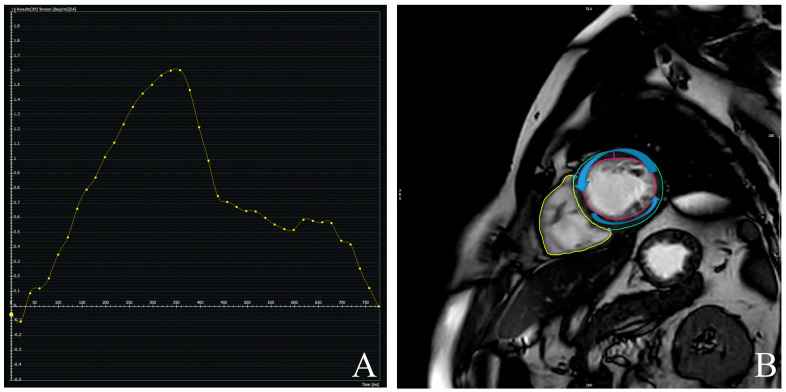
Example of left ventricular torsion (**A**) assessment using feature-tracking cardiac magnetic resonance (FT-CMR). Short-axis planes are tracked throughout the cardiac cycle, and rotational displacement is quantified (**B**). Left ventricular torsion is derived as the rotation difference, normalized to ventricular length.

GLS and peak systolic LV torsion were calculated using CMR-FT analysis (cvi42, version 6.0.0), following published consensus protocols [25]. GLS was computed from long-axis (2-, 3-, and 4-chamber) cine views (Figure 2). Endocardial and epicardial borders were manually delineated at end-diastole, with automated tracking throughout the cardiac cycle. LV torsion (Figure 3) was defined as the difference in rotational angle between apical and basal short-axis planes at peak systole, divided by the longitudinal distance between them and expressed in degrees per centimeter.

Complementary functional markers included LV longitudinal axis shortening (LV-LAS) and the LV sphericity index (LVSI), both derived from standard cine images. Segments with poor tracking quality or artifacts were excluded. Papillary muscles, trabeculations, pericardial structures, and epicardial fat were excluded from all contour analyses.

Reproducibility was evaluated in a random subset of 18 patients representative of the overall cohort. Intra- and inter-observer variability were assessed for key functional parameters, including GLS, GCS, GRS, and LV torsion. Two independent observers, blinded to all clinical and imaging data, each performed two measurements per case.

### 2.3. Clinical Follow-Up and Outcome Definition

All patients were monitored over a median period of 33 months (interquartile range: 4–64 months) using a structured follow-up strategy that combined periodic clinical evaluations and telephone-based assessments. The primary outcome was defined as the first occurrence of a major adverse cardiovascular event (MACE), comprising cardiac death, sustained ventricular arrhythmias, or heart failure hospitalization. Events unrelated to cardiovascular pathology were not included in the analysis.

### 2.4. Statistical Analysis

Continuous variables were reported as mean ± standard deviation (SD) or median with interquartile range (IQR), and categorical variables as counts and percentages. Group comparisons were performed using Student’s *t*-test or Mann–Whitney U test for continuous variables and chi-square or Fisher’s exact test for categorical data. Survival analysis was conducted using Kaplan–Meier curves and the log-rank test. Cox proportional hazards models were applied to identify independent predictors of major adverse cardiovascular events (MACEs). The multivariable Cox model included four predictors and was based on 24 primary events, resulting in an events-per-variable (EPV) ratio of 6.0; this potential risk of overfitting was considered when interpreting the results. A two-tailed *p*-value < 0.05 was considered statistically significant. All analyses were performed using SPSS version 26.0 and MedCalc version 20.305.

## 3. Results

### 3.1. Baseline Characteristics

The final cohort comprised 150 patients with DCM and 100 healthy volunteers (Table 1). There were no significant differences in age (mean 52 vs. 53 years), sex distribution, or body mass index (27.6 vs. 28.5 kg/m^2^, *p* = NS). The prevalence of hypertension, diabetes mellitus, and dyslipidemia was similar between groups. However, DCM patients exhibited significantly higher resting heart rate (74 vs. 72 bpm, *p* < 0.05) and systolic blood pressure (132 vs. 109 mmHg, *p* < 0.05). New York Heart Association (NYHA) functional class I/II/III distribution among DCM patients was 28/51/22, reflecting a spectrum of functional impairment. The use of beta-blockers and renin-angiotensin system inhibitors was significantly more common in the DCM group (*p* < 0.05 for both).

Intraclass correlation coefficients (two-way random-effects model, absolute agreement) and Cohen’s kappa values were calculated. For intra-observer reproducibility, the same observer repeated the measurements after approximately one week. Intra-observer kappa values were 0.92 for GLS, 0.90 for GCS, and 0.91 for LV torsion, while inter-observer values were 0.86, 0.87, and 0.85, respectively. All parameters demonstrated excellent reproducibility (ICC > 0.89), supporting the robustness of CMR-FT–derived metrics and indicating minimal influence of measurement variability on study findings.

### 3.2. CMR-Derived Structural and Functional Parameters

CMR revealed marked differences in ventricular structure and function between groups (Table 2). Patients with DCM had significantly increased LVEDVi (134.6 vs. 63.3 mL/m^2^), LV end-systolic volume index (LVESVi: 90.7 vs. 22.1 mL/m^2^), and LV mass index (LVMi: 87.4 vs. 58.8 g/m^2^), all with *p* < 0.001. LVEF was notably reduced in the DCM group (35.1% vs. 65.2%, *p* < 0.001).

**Figure 4:**
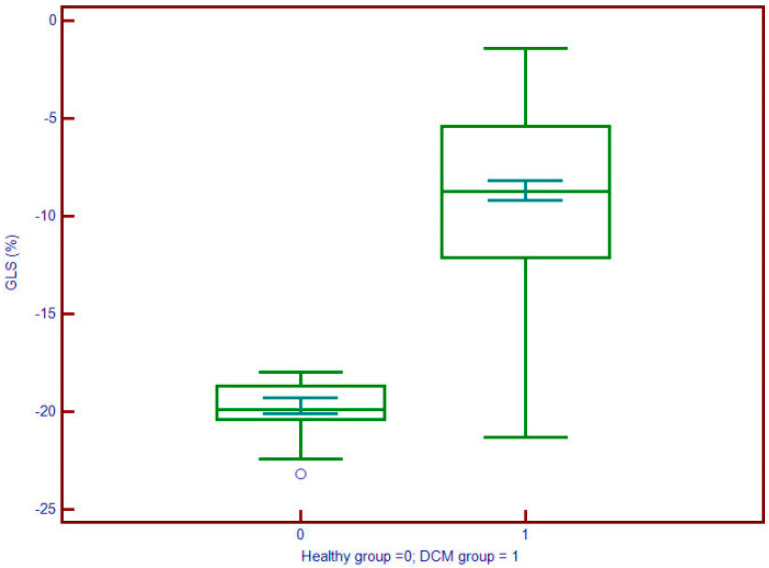
Global longitudinal strain (GLS) values are significantly less negative in patients with DCM (group 1; median ≈ –10.5%) compared to controls (group 0; median ≈ –18%), indicating impaired myocardial deformation. The interquartile range in DCM patients (approx. –12% to –8%) is clearly shifted toward dysfunction, supporting the utility of GLS in identifying subclinical systolic impairment.

**Figure 5:**
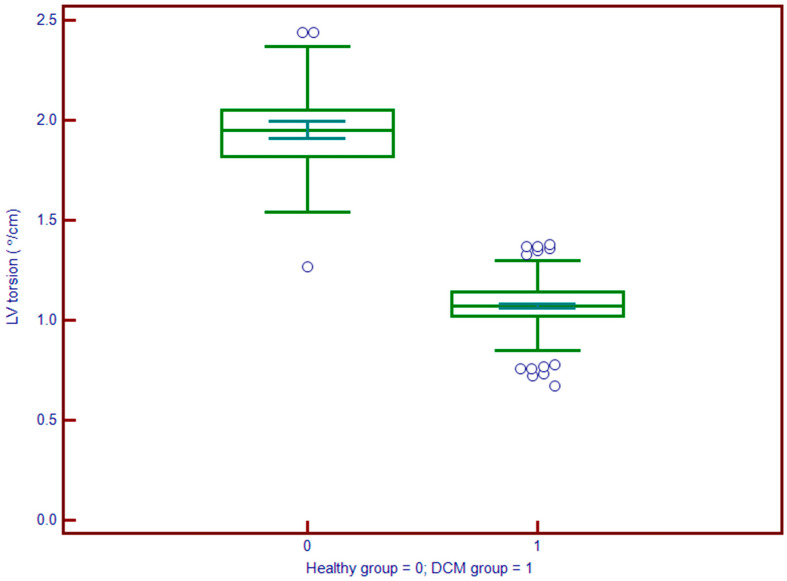
Left ventricle (LV) torsion is significantly reduced in patients with DCM (group 1; median ≈ 5°) compared to controls (group 0; median ≈ 12°). The marked shift in the distribution indicates impaired left ventricular twist mechanics in DCM, underscoring the role of torsion as a sensitive marker of early systolic dysfunction.

Left atrial volume index (LAVi) was also significantly elevated in DCM (57.8 vs. 30.6 mL/m^2^, *p* < 0.001). Myocardial deformation parameters showed significant impairment in DCM. GLS (Figure 4) was substantially reduced (mean: −9.2% vs. −19.7%, *p* < 0.001), and LV torsion was likewise diminished (1.04 vs. 1.95 °/cm, *p* < 0.001) (Figure 5). These results underscore profound alterations in both systolic function and myocardial mechanics.

### 3.3. Prognostic Value of CMR-Derived GLS

GLS emerged as a strong predictor of cardiovascular outcomes. In univariable Cox regression, reduced GLS was associated with increased MACE risk (hazard ratio [HR]: 1.21; 95% confidence interval [CI]: 1.01–1.44; *p* = 0.034) Table 3. This association remained significant after adjustment for conventional risk factors (adjusted HR: 1.09; 95% CI: 1.01–1.61; *p* < 0.01). Patients with GLS below the median (≤−8.6%) demonstrated significantly shorter event-free survival. These findings support the clinical utility of CMR-derived GLS in risk stratification for patients with DCM.

### 3.4. Prognostic Value of CMR-Derived LV-Torsion

**Figure 6:**
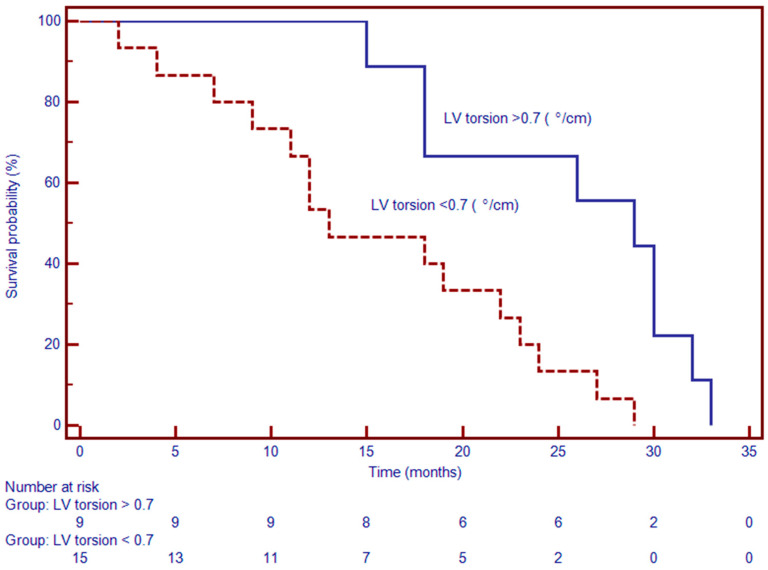
Kaplan–Meier survival curves demonstrate significantly worse event-free survival in patients with myocardial torsion < 0.7°/cm compared to those with torsion ≥ 0.7°/cm. At 24 months, approximately 70% of patients with reduced torsion remained event-free, versus over 90% in the preserved torsion group (*p* < 0.001). The survival curves begin to diverge early and remain separated over the observation period, indicating a strong association between impaired torsion mechanics and adverse clinical outcomes.

LV torsion was an independent prognostic marker (Figure 6). Patients with torsion values below the median experienced a higher rate of MACE during follow-up. In multivariable analysis, lower LV torsion was independently associated with increased event risk (HR: 1.37; 95% CI: 1.03–1.81; *p* < 0.01) Table 3. These results highlight the additive value of myocardial rotational mechanics in prognostication, beyond conventional volumetric and functional indices.

### 3.5. Association of Myocardial Mechanics with LGE

**Figure 7:**
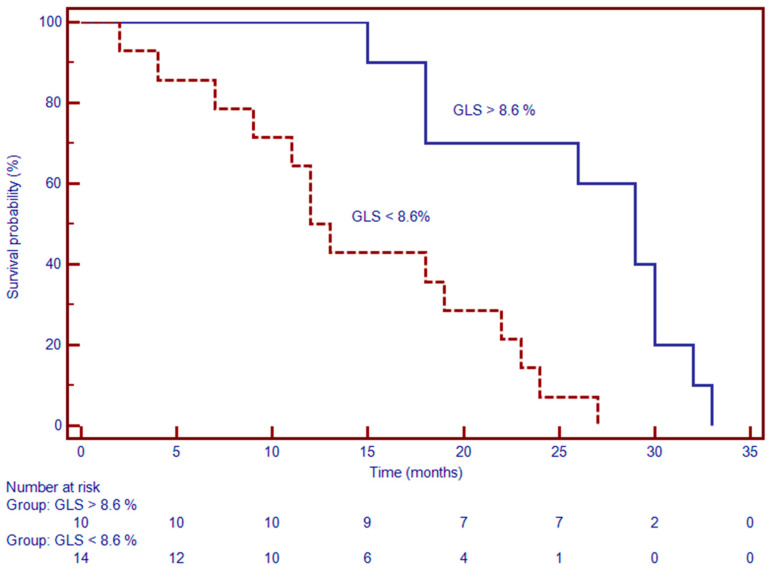
Kaplan–Meier survival analysis stratified by GLS threshold (–13%) reveals significantly worse event-free survival in patients with GLS > –13% (i.e., more impaired strain). At 24 months, event-free survival was approximately 68% in the impaired GLS group, compared to over 90% in patients with GLS ≤ –13% (*p* < 0.001). The early and persistent divergence of the curves underscores the prognostic significance of impaired GLS on the outcome.

LGE was present in 42% of DCM patients and was significantly associated with adverse outcomes. Kaplan–Meier analysis revealed a median event-free survival of 13 months in patients with LGE versus 29 months in those without. In Cox regression, LGE positivity was linked to a substantially elevated risk of MACE (HR: 2.86; 95% CI: 1.48–12.52; *p* < 0.001) (Figure 7). These data affirm the prognostic significance of myocardial fibrosis as identified by contrast-enhanced CMR. Together, these findings suggest that CMR-derived measures of myocardial deformation and tissue characterization—particularly GLS, LV torsion, and LGE—offer powerful, independent prognostic information in patients with DCM.

## 4. Discussion

The current article underlines the independent and complementary prognostic significance of CMR-derived GLS and LV torsion in patients with DCM. Our findings align with and expand upon existing literature, emphasizing the potential of these parameters in enhancing risk stratification and improving prognosis prediction.

GLS has emerged as a sensitive marker for detecting subclinical myocardial dysfunction, often preceding noticeable declines in LVEF. In our cohort, a GLS value ≤ −8.6% was significantly associated with shorter event-free survival. This observation is consistent with previous studies demonstrating the prognostic utility of GLS in DCM. For instance, a multicenter study involving 350 DCM patients reported that impaired CMR-determined GLS was a strong predictor of adverse outcomes, providing incremental predictive value beyond LVEF and LGE [26]. Furthermore, a recently published meta-analysis which included 6 studies highlighted the incremental value of GLS over traditional parameters in forecasting mortality and heart failure hospitalization in DCM patients. It was shown that the highest predictive ability of GLS was in those with LVEF over 30% [18].

LV torsion reflects the helical arrangement of myocardial fibers and is integral to efficient cardiac mechanics. Our study identified reduced LV torsion as an independent predictor of MACE, underscoring its prognostic relevance. This aligns with findings from another recently published work that demonstrated the association of impaired LV torsion with increased risk of cardiac events in DCM patients [26]. Additionally, research has shown that LV torsion provides incremental prognostic information beyond LVEF and LGE in various cardiac pathologies [27,28]. Further studies still need to be conducted to validate the clinical utility of LV torsion in day-to-day practice.

Myocardial fibrosis, as detected by LGE, disrupts the structural integrity and contractile function of the myocardium. In our study, the presence of LGE was significantly associated with impaired GLS and LV torsion, as well as with adverse clinical outcomes. This corroborates previous findings that link myocardial fibrosis to deteriorated myocardial mechanics and increased risk of arrhythmias and mortality in DCM patients [26]. Moreover, the integration of strain parameters with LGE has been shown to enhance risk stratification, facilitating the identification of high-risk individuals who may benefit from more aggressive therapeutic interventions [29,30].

Beyond DCM, GLS and LV torsion have demonstrated prognostic value across a spectrum of cardiovascular diseases. In patients with ST-elevation myocardial infarction (STEMI), reduced LV torsion measured by CMR has been associated with adverse remodeling and increased incidence of major adverse cardiac and cerebrovascular events [11]. Similarly, GLS has been identified as a predictor of mortality and heart failure hospitalization in conditions such as hypertrophic cardiomyopathy, myocarditis, and aortic stenosis [7,31,32,33]. These findings highlight the versatility and clinical utility of myocardial deformation parameters in diverse cardiac pathologies.

The integration of CMR-derived GLS and LV torsion into routine clinical practice could offer a more nuanced assessment of subclinical myocardial dysfunction, enabling early identification of patients at elevated risk for adverse outcomes. Our findings support the clinical utility of these parameters by adopting them into day-to-day practice. Nevertheless, future research should focus on establishing standardized thresholds for GLS and LV torsion, exploring their role in guiding therapeutic decisions, and validating their prognostic utility in larger, multicenter cohorts.

Study Limitations: This single-center study may limit the generalizability of our findings. Although CMR-FT is a valuable tool for assessing myocardial function, its accuracy can be influenced by arrhythmias or suboptimal image quality, potentially affecting GLS and LV torsion measurements. The study’s observational design precludes causal inference. The relatively small number of primary events (n = 24) resulted in an events-per-variable ratio of 6.0, which may have reduced statistical power and increased the risk of overfitting. Furthermore, the absence of advanced tissue characterization techniques such as T1/T2 mapping and ECV quantification limited the evaluation of diffuse myocardial fibrosis and its relationship to deformation parameter. The exclusion of comorbidities in the control group may reduce the generalizability of our findings, as this group may not reflect the broader population where such comorbidities are prevalent, potentially leading to an overestimation of differences between patients and controls.

## 5. Conclusions

Our findings endorse that CMR-derived GLS and LV torsion might become strong, independent predictors of adverse outcomes in patients with DCM. These findings support the integration of GLS and torsion into routine CMR protocols, enabling earlier and more personalized clinical decision-making. Future prospective studies are warranted to validate the prognostic value of strain parameters and their integration into multimodal risk assessment models for clinical decision-making.

## Figures and Tables

**Table 1:** Baseline characteristics of patients in study.

	DCM Group n = 150	Healthy Group n = 100	*p*-Value
Clinical characteristics			
- Age, mean (SD), years	52 (14.2)	53 (12.1)	NS
- Male sex, n (%)	110 (73.3)	61 (61)	NS
- Body-mass index, kg/m^2^	27.6 (4.8)	28.5 (4.3)	NS
- Heart rate, mean (SD), bpm	74 (15.8)	72 (9.3)	<0.05
- Systolic blood pressure, mean (SD), mmHg	132 (19.1)	109 (12.9)	<0.05
- Hypertension, n (%)	79 (52.6)	51 (51)	NS
- Diabetes mellitus, n (%)	45 (30)	29 (29)	NS
- Dyslipidemia, n (%)	86 (57.3)	48 (48)	NS
- Smoking, n (%)	48 (32)	26 (26)	NS
- NYHA functional class I/II/III, n	28/51/22	15/0/0	NS
Electrocardiogram			
- Atrial fibrillation, n (%)	20 (13.3)		NA
- Left bundle branch block, n (%)	14 (9.3)	5 (5)	NS
- Right bundle branch block, n (%)	14 (9.3)	4 (4)	NS
- Significant Q waves, n (%)	17 (11.3)		NA
Medications			
- Beta-blockers, n (%)	130 (86.6)	30 (30)	<0.05
- ACEIs or ARBs, n (%)	113 (75.3)	37 (37)	<0.05
- Calcium channel blockers, n (%)	8 (5.3)	2 (2)	NS
- Statins, n (%)	87 (58)	63 (63)	NS
- Antiplatelet therapy, n (%)	52 (34.6)		NS
- Diuretics, n (%)	96 (64)	54 (54)	NS
Biomarker levels			
- NT-proBNP, median (IQR), pg/mL	2807 (570–10,870)	214 (60–370)	NS
- eGFR, mean (SD), mL/min/1.73 m^2^	81.9 (17.2)	86.8 (21.1)	NS

Abbreviations: ACEI, angiotensin-converting enzyme inhibitor; ARB, angiotensin receptor blocker; bpm, beats per minute; DCM, dilated cardiomyopathy; IQR, interquartile range; n, number of patients; NT-proBNP, N-terminal pro-Brain Natriuretic Peptide; NYHA, New York Heart Association; SD, standard deviation.

**Table 2:** cMRI parameters between healthy and DCM group.

	DCM Group n = 150	Healthy Group n = 100	*p*-Value
Cardiovascular magnetic resonance			
- LVEDV index, mean (SD), mL/m^2^	134.6 (35.5)	63.3 (17.2)	<0.001
- LVESV index, mean (SD), mL/m^2^	90.7 (34.8)	22.1 (7.6)	<0.001
- LVM index, mean (SD), g/m^2^	87.4 (20.6)	58.8 (12.6)	<0.001
- LVEF, mean (SD), %	35.1 (9.5)	65.2 (5.2)	<0.001
- LAV index, mean (SD), mL/m^2^	57.8 (21.7)	30.6 (12.2)	<0.001
- GLS, mean (SD), %	−9.2 (4.5)	−19.7 (1.3)	<0.001
- LV torsion mean (SD), °/cm	1.04 (0.19)	1.95 (0.22)	<0.001
- LVSI, mean (SD)	0.41 (0.10)	-	NA
- LV-LGE, n (%)	63 (42)	-	NA
- LV-LGE mass median (IQR), g	34.0 (4–82)	-	NA
- RVEDV index, mean (SD), mL/m^2^	54.2 (22.4)	56.5 (23.2)	NS
- RVESV index, mean (SD), mL/m^2^	29.4 (15.5)	22.2 (20.0)	<0.01
- RVEF, mean (SD), %	45.9 (9.7)	60.7 (5.2)	<0.01

Abbreviations: DT, early diastolic filling deceleration time; E, peak mitral flow velocity; E’, early diastolic peak myocardial velocity; IQR, interquartile range; LAS, left ventricular longitudinal-axis strain; LAV, left atrial volume; LGE, left ventricular late gadolinium enhancement; LVEF, left ventricular ejection fraction; LVEDV, left ventricular end-diastolic volume; LVESV, left ventricular end-systolic volume; LVM, left ventricular mass; LVSI, left ventricular sphericity index; n, number of patients; RVEDV, right ventricular end-diastolic volume; RVEF, right ventricular ejection fraction; RVESV, right ventricular end-systolic volume; SD, standard deviation; sPAP, systolic pulmonary artery pressure; TAPSE, tricuspid annular plane systolic excursion. Data are reported as mean (standard deviation) or median (IQR) or n (%).

**Table 3:** Univariable and multivariable Cox analysis testing between studied parameters and MACEs.

	No MACE n = 126	MACE n = 24	Univariable Analysis		Multivariable Analysis	
			Unadjusted HR (95% CI)	*p* Value	Adjusted HR (95% CI)	*p* Value
Age, years	52 (13.4)	51 (17.9)	1.00 (0.98–1.02)	NS		
Male sex, n, %	92 (73.0)	18 (75.0)	1.11 (0.53–2.48)	NS		
Body-mass index, kg/m^2^	28.1 (4.7)	24.9 (4.7)	1.00 (0.94–1.12)	NS		
Systolic blood pressure	132 (19.5)	135.8 (16.6)	0.97 (0.95–1.00)	NS		
NT-proBNP, pg/mL	2729 (600–10,870)	2919 (570–9900)	0.96 (0.998–1.02)	NS		
LVEDV index, mL/m^2^	133.9 (35.6)	138.8 (35.3)	1.01 (0.98–1.01)	NS		
LVESV index, mL/m^2^	89.2 (34.7)	98.6 (35.5)	0.99 (0.98–1.01)	NS		
LVM index, g/m^2^	87.8 (20.2)	85.5 (22.8)	1.01 (0.98–1.03)	NS		
LVEF, %	34.8 (9.2)	30.4 (10.1)	1.02 (0.96–1.47)	NS		
LAV index, mL/m^2^	56.2 (21.1)	66.4 (23.2)	0.99 (0.97–1.01)	NS		
GLS, %	−9.5 (4.8)	−7.7 (2.1)	1.21 (1.01–1.44)	0.034	1.09 (1.01–1.61)	<0.01
LV torsion, °/cm	1.04 (0.2)	1.03 (0.15)	1.44 (1.03–2.00)	0.029	1.37 (1.03–1.81)	<0.01
LGE +, n	48 (38.1)	15 (62.5)	4.23 (1.48–12.52)	0.007	2.86 (0.00–12.51)	<0.001
LVSI, %	0.41 (0.10)	0.42 (0.11)	1.13 (1.01–2.02)	0.047	0.87 (0.79–1.37)	NS
RVEDV index, mL/m^2^	52.8 (20.9)	55.5 (29.8)	1.03 (0.99–1.02)	NS		
RVESV index, mL/m^2^	28.4 (13.6)	34.8 (26.6)	1.01 (0.99–1.02)	NS		
RVEF, %	46.8 (9.3)	41.2 (10.5)	0.95 (0.92–1.01)	NS		

Abbreviations: E, peak mitral flow velocity; E’, early diastolic peak myocardial velocity; eGFR, estimated glomerular filtration rate; IQR, interquartile range; LAS, left ventricular longitudinal-axis strain; LAV, left atrial volume; LGE, left ventricular late gadolinium enhancement; LVEF, left ventricular ejection fraction; LVEDV, left ventricular end-diastolic volume; LVESV, left ventricular end-systolic volume; LVM, left ventricular mass; LVSI, left ventricular spherical index; n, number of patients; NT-proBNP, N-terminal pro-Brain Natriuretic Peptide; RVEDV, right ventricular end-diastolic volume; RVEF, right ventricular ejection fraction; RVESV, right ventricular end-systolic volume; TAPSE, tricuspid annular plane systolic excursion.

## Data Availability

The original contributions presented in this study are included in the article. Further inquiries can be directed to the corresponding author.
